# An increase in immature β-cells lacking Glut2 precedes the expansion of β-cell mass in the pregnant mouse

**DOI:** 10.1371/journal.pone.0182256

**Published:** 2017-07-28

**Authors:** Christine A. Beamish, Linhao Zhang, Sandra K. Szlapinski, Brenda J. Strutt, David J. Hill

**Affiliations:** 1 Department of Physiology and Pharmacology, Western University, London, ON, Canada; 2 Children’s Health Research Institute, London, ON, Canada; 3 Lawson Health Research Institute, St Joseph Health Care, London, ON, Canada; 4 West China School of Medicine, Sichuan University, Chengdu, Sichuan, China; 5 Department of Medicine, Western University, London, ON, Canada; University of Michigan, UNITED STATES

## Abstract

A compensatory increase in β-cell mass occurs during pregnancy to counter the associated insulin resistance, and a failure in adaptation is thought to contribute to gestational diabetes. Insulin-expressing but glucose-transporter-2-low (Ins^+^Glut2^LO^) progenitor cells are present in mouse and human pancreas, being predominantly located in extra-islet β-cell clusters, and contribute to the regeneration of the endocrine pancreas following induced ablation. We therefore sought to investigate the contribution of Ins^+^Glut2^LO^ cells to β-cell mass expansion during pregnancy. Female C57Bl/6 mice were time mated and pancreata were collected at gestational days (GD) 6, 9, 12, 15, and 18, and postpartum D7 (n = 4/time-point) and compared to control (non-pregnant) animals. Beta cell mass, location, proliferation (Ki67^+^), and proportion of Ins^+^Glut2^LO^ cells were measured using immunohistochemistry and bright field or confocal microscopy. Beta cell mass tripled by GD18 and β-cell proliferation peaked at GD12 in islets (≥6 β-cells) and small β-cell clusters (1–5 β-cells). The proportion and fraction of Ins^+^Glut2^LO^ cells undergoing proliferation increased significantly at GD9 in both islets and clusters, preceding the increase in β-cell mass and proliferation, and their proliferation within clusters persisted until GD15. The overall number of clusters increased significantly at GD9. Quantitative PCR showed a significant increase in Pdx1 presence at GD9 *vs*. GD18 or control pancreas, and Pdx1 was visualized by immunohistochemistry within both Ins^+^Glut2^LO^ and Ins^+^Glut2^HI^ cells within clusters. These results indicate that Ins^+^Glut2^LO^ cells are likely to contribute to β-cell mass expansion during pregnancy.

## Introduction

An adaptive increase in maternal pancreatic β-cell mass occurs during pregnancy in response to circulating placental hormones and an increased insulin resistance in adipose tissue, skeletal muscle, and liver [1]. In the mouse a two-to-three-fold increase in β-cell mass occurs largely as a result of increased β-cell proliferation which is maximal around gestational day (GD) 12 [2]. Van Assche et al [3] reported that the fractional area of β-cells within the pancreas was increased two-fold in pregnant women who suffered accidental death in third trimester, or who died at parturition, compared to age-matched non-pregnant control cases. A subsequent report from Butler et al [4] found a 40% increase in the fractional area of β-cells during pregnancy at post mortem, although this study included subjects who died during first trimester when the increase in β-cell mass was likely to be sub-maximal. Failure to undergo such functional adaptations has been linked with an increased risk of gestational diabetes [5]. Following parturition a regression of β-cells occurs, with a return to the original pre-pregnancy mass within 10 days through a wave of apoptosis.

The maternal peripheral insulin resistance of pregnancy is due, in part, to the actions of variant growth hormone (GH-V) released into maternal blood from the placental syncytiotrophoblast [6]. Both GH-V and placental lactogen (PL) also increase maternal hepatic gluconeogenesis and lipolysis to maintain trans-placental transport to the fetus. These diabetogenic stimuli are countered by a re-activation of β-cell proliferation by increasing levels of PL [7]. An over-expression of PL in mouse β-cells resulted in increased proliferation [8]. The ability of PL to overcome β-cell quiescence and re-activate mitosis is mediated by the interaction of PL with prolactin receptors, since deletion of the prolactin receptor on β-cells abolished compensatory growth during pregnancy, leading to impaired insulin release and glucose intolerance [9, 10]. Over-expression of the prolactin receptor resulted in β-cell expansion [10]. Subsequent to PL binding to prolactin receptors, there is an activation of the Jak2/Stat5 signaling pathways, including mitogen-activated protein kinases (MAPK), PI3K, and Akt, resulting in β-cell proliferation [11, 12]. However, a number of other locally-released peptides have also been implicated in β-cell expansion during pregnancy including islet-derived glucagon-like peptide 1 (GLP-1) [13], survivin [14], and hepatocyte growth factor (HGF) [15].

The adaptive increase in β-cell mass during pregnancy may also result from the differentiation of resident pancreatic progenitor cells. When insulin-expressing β-cells were genetically tagged in mouse the abundance of the lineage-marked cells was shown to be diluted in the pancreas during pregnancy, suggesting that new β-cells were generated, in part, from non-insulin-expressing progenitors [16, 17]. The ‘new’ β-cells were more abundant in small extra-islet endocrine clusters than within the larger islets [16], and particularly those adjacent to the pancreatic ducts, suggesting that neogenesis may have occurred from ductal progenitors. The abundance of Ngn3-expressing cells was found to be increased 3.5–fold by day 14 of mouse pregnancy by Sostrup et al. [18] but within the acinar tissue, suggesting acinar as a location for progenitors. Contrary findings were reported by Toselli et al. [17] who showed that the population of hormone-negative/neurogenin3 (Ngn3)-positive cells were mainly within islets.

Outside of pregnancy multi-lineage potential progenitors with the capacity to become β-cells have been identified in both the mouse and human pancreas [19]. Smukler et al. [20] reported a population of cells that expressed insulin in small amount, but exhibited a negative-to-low expression of the glucose transporter, Glut2 such that they were unresponsive to glucose stimulation of insulin release. However, these cells could differentiate in vitro to become mature, functional β-cells, as reviewed recently [21]. We demonstrated using flow cytometry and immunofluorescence that such multi-lineage potential Ins^+^Glut2^LO^ cells were abundant within small extra-islet pancreatic β-cell clusters (≤5 β-cells) relative to larger islets in both mouse and human pancreas [22, 23], and decreased with age [23]. The activation of such resident progenitors with a partial β-cell phenotype could potentially contribute to an increased β-cell mass during pregnancy without the need for trans-differentiation from ductal epithelium or acinar tissue. Therefore, we have examined the altered abundance, location and proliferation of resident pancreatic Ins^+^Glut2^LO^ cells throughout mouse pregnancy.

## Materials and methods

### Animals

Adult (8 week old) C57BL/6 mice were obtained from Charles River Laboratories (Wilmington, MA) and housed at the Lawson Health Research Institute, London, ON, Canada. Timed pregnancies were accomplished by establishing mouse estrous cycling using vaginal cytology according to Byers et al [24]. The morning of pro-estrus, individual nulliparous female and male mice were housed together for mating. Day zero of pregnancy was determined the following morning by identification of sperm under light microscope or a vaginal plug. Males were removed from the cage once mating was confirmed. Pregnant mice were weighed daily and were sacrificed by CO_2_ asphyxia at gestational days (GD) 6, 9, 12, 15, 18, or 7 days postpartum (PP D7) (n = 4 per time point) for comparison to control (non-pregnant) age-matched female mice (n = 4). All animals were housed under a 12-hour light/dark cycle and received food and water ad libitum. This study on an increase in immature β-cells lacking Glut2 that precedes the expansion of β-cell mass in the pregnant mouse received prior approval from the Animal User Committee, Western University and adheres to the Guidelines of the Canadian Council for Animal Care. Mice were euthanized by exposure to CO_2_ in an approved chamber.

Pancreata were dissected immediately following sacrifice and placed directly into 4% paraformaldehyde (PFA, Electron Microscopy Sciences, Hatfield, PA). Fixed mouse tissue was prepared and sectioned along the longitudinal axis of the pancreas according to Beamish et al [22]. Three cryosections were cut from each pancreas, each of seven microns thickness and representing at least 2 longitudinal areas of the pancreas, with an interval between each layer > 150 μm. The coefficient of variation between replicate sections was found to be 8% when calculating mean beta cell area. Slides were stored at -20°C.

### Immunohistochemical analysis

Immunohistochemistry was performed as described by Beamish et al [22]. Cryosections were immunostained using antibodies against insulin (1:200, Santa Cruz, Santa Cruz, TX), Glut2 (1:200, Santa Cruz), or mouse Ki67 antigen (1:50 BD Biosciences, Mississauga, ON, Canada), and secondary antibodies (1:500, Life Sciences) were directed against the primary antibody using 555, 488, and 647 fluorophores, respectively. Heat induced antigen retrieval was required for Ki67 and accomplished using 100 mM Tris-EDTA at 100°C for 30 min in a decloaking chamber. DAPI (4, 6-diamidino-2 phenylindole, dihydrochloride) (1/500, D1306) was used as a nuclear counterstain for cell counting. Three sections from separate regions of each pancreas were analyzed for immunofluorescence. Slides were viewed under a Zeiss 510 LSM confocal microscope at the Biotron, Western University, Canada and cell counting analysis was performed using the LSM Image Browser (Zeiss). Every insulin-expressing cell was imaged per section, with inclusion criteria as ≥ 300 insulin^+^ cells from at least 10 islets per slide (average 792 β-cells from 18 islets counted per slide). All extra-islet β-cell-containing clusters were counted per section (average 32 clusters containing 59 insulin-expressing cells. In this model, an “islet” was considered to contain > 5 insulin-expressing cells, and an extra-islet “cluster” as containing between 1–5 β-cells. Co-staining with Ki67 and the visualization of a nuclear localization was used as a measurement of insulin-immunopositive cells undergoing proliferation. The percentage of such cells was calculated using the sample sizes stated above for all insulin-expressing cells identified within either islets or clusters for each section. These were then further separated into sub-populations of Ins^+^Glut2^LO^ or Ins^+^Glut2^HI^ cells based on the presence of Glut2 as visualized by co-immunostaining.

Beta cell mass was calculated from three sections as described by Chamson-Reig et al [25], using a mouse anti-insulin primary antibody (Sigma Chemical, St Louis, MO; 1/2000), biotinylated horse anti-mouse secondary antibody (Vector Laboratories, Burlington, Canada), and DAB (diaminobenzoate) chromagen (Biogenex Inc., Fremont, CA). The entire pancreas section was imaged at 2.5X magnification and insulin-positive cells were imaged at 40X magnification, and three tissue sections were analyzed per pancreas. Beta cell area was calculated by dividing insulin-positive cell area by total pancreas sectional area after tracing and analyzed using Northern Eclipse software (v. 6.0, Empix Imaging, Mississauga, ON, Canada). Beta-cell mass was calculated by multiplying beta cell area by the weight of the pancreas.

### Glucose tolerance tests

Intra-peritoneal glucose tolerance tests (IPGTT) were performed on control (non-pregnant) and pregnant (GD9 and GD18) female mice. Mice were fasted for 4 h, then injected with 5 ul/g body weight of 40% glucose solution, and blood glucose measured from the tail at 0, 5, 15, 30, 60, 90, and 120 min using a One Touch Ultra glucometer (Lifescan Inc., Milpitas, CA). Mice were euthanized and blood aspirated from the heart. Blood was centrifuged ≥ 2000 x g for 10 min, and serum frozen at -20°C. Insulin content in serum was measured using an RIA (St. Joseph’s Health Care, London, ON, Canada), and compared between groups.

### Quantitative polymerase chain reaction

Whole pancreas from GD9, GD18, and non-pregnant mice were dissected after GTT. Each pancreas was cut into 4 pieces and placed in 2 ml RNAlater solution, and frozen at -20C. Samples were minced with scissors in lysis buffer and Qiashredders (Qiagen, 79564) prior to total RNA extraction using RNeasy Plus Mini kits (Qiagen, 74034). Total RNA (<1 μg) was extracted and reverse transcribed using iScript Reverse Transcription Supermix (Bio-Rad Laboratories, 170–8840). Quantitative PCR experiments were accomplished using the 2^-ΔΔC^_T_ method after confirmation of parallel PCR amplification efficiencies between each gene of interest and one of two housekeeping genes on a Real-Time PCR ABI Prism 7500. IQ SYBR Green (Bio-Rad, 170–8882) was employed for detection of PCR products, with primer sequences provided in Table 1. PCR reactions were run in duplicate and performed using an initial denaturation at 95°C for 5 min, followed by cycles of denaturation (95°C for 15 sec), primer annealing (60°C for 1 min) and transcript extension (50°C for 2 min) for 40 cycles. Levels of mRNA expression were calculated relative to those of the housekeeping gene cyclophilin A (cycloA) for V-maf musculoaponeurotic fibrosarcoma oncogene homolog B (MafB), and Glyceraldehyde 3-phosphate dehydrogenase (GAPDH) for pancreatic duodenal homeodomain box 1 (Pdx1) and homeobox protein Nkx_6.1(Nkx6.1).

**Table 1:** Primer sequences designed for the amplification of mouse cDNA by qPCR.

Gene	Sense primer (5’-3’)	Antisense Primer (5’-3’)	Band Size (bp)
Pdx1	gttggatagccggagagatg	agtttggagcccaggttg	151
Nkx6.1	tctggctgtgggatgttagc	tcatctcggccatactgtgc	106
MafB	ggtataaacgcgtccagcag	cgagtttctcgcacttgacc	138
CycloA	atggtcaaccccaccgtgt	ttctgctgtctttggaactttgtc	102
GAPDH	accatcttccaggagcgaga	ctcgtggttcacacccatca	192

### Measurement of Pdx1

Mouse pancreas Pdx1 protein was measured by ELISA (Abbexa Ltd, Cambridge, UK) within the supernatant of sonicated pancreas at a dilution of 1:100 and expressed relative to total protein content.

### Statistical analysis

Data are presented as mean ± SEM, with statistics analyzed using GraphPad Prism software (v5.1, San Diego, CA, USA). Normality of data distribution was determined using the Shapiro-Wilk test. For the majority of experiments students t-tests or one-way ANOVA with Tukey’s post-test and Bartlett’s test for equal variance were used for comparison between study groups and at each time point. For non-parametric data, ANOVA with Dunn’s multiple comparison test was used with Kruskal-Wallis assessment. For comparison of gene-specific mRNA levels between non-pregnant mouse and pregnant mouse pancreas at GD9 or 18, fold changes were compared using a t-test with Mann-Whitney rank score. Significance was determined as p < 0.05.

## Results

### Beta-cell mass and glycemic control reflect changes associated with pregnancy

**Fig 1:**
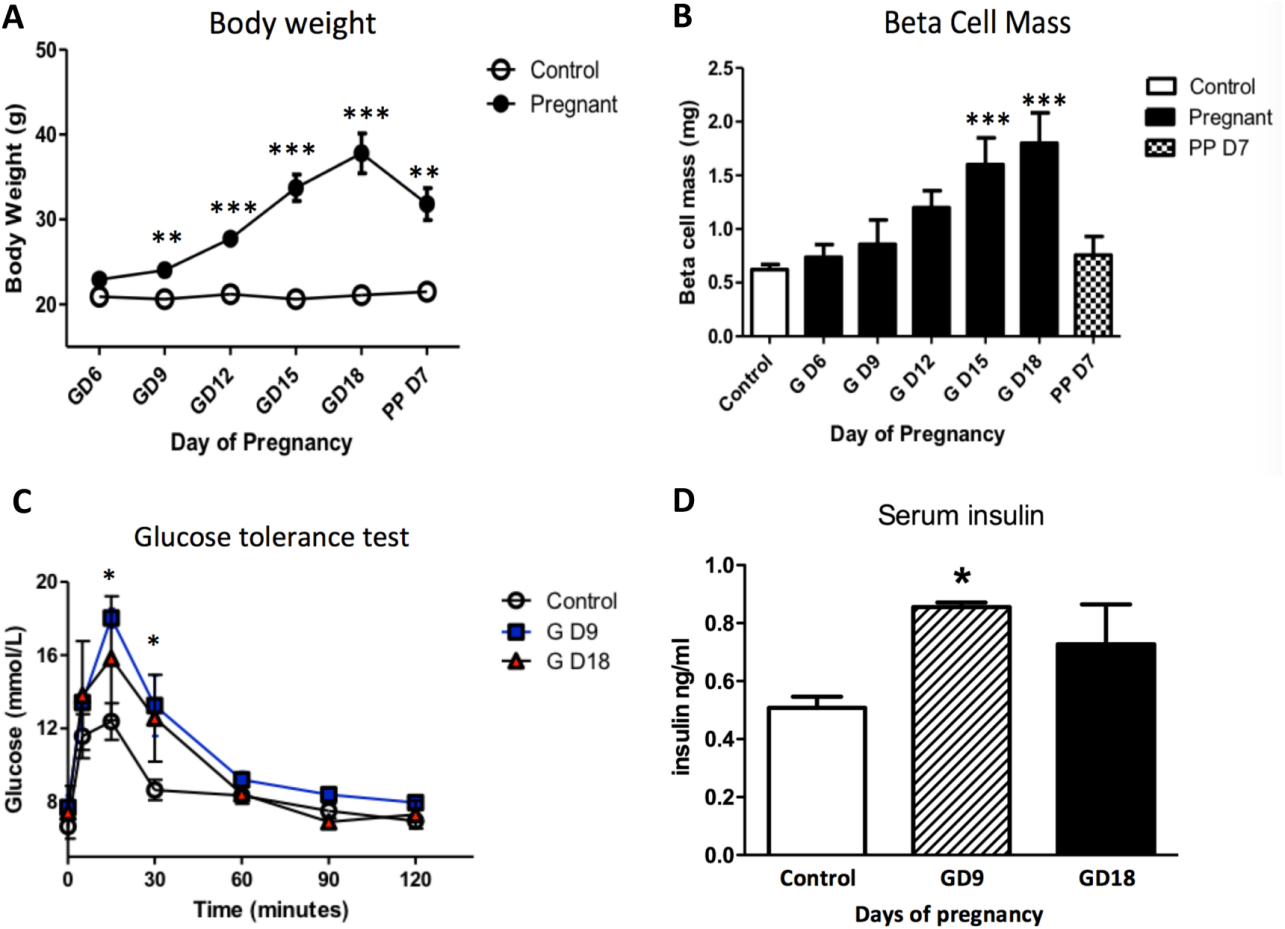
**Changes in maternal body weight (A), β-cell mass during pregnancy (GD—gestational day) and at postpartum day 7 (PP D7) (B), glucose tolerance (C) and serum insulin levels (D) at GD9 and 18 compared to non-pregnant control mice (n = 5 per time point).** Maternal β-cell mass increased through gestation and peaked at GD15 to 18, before declining postpartum. A relative glucose intolerance was associated with the development of pregnancy despite the increasing in β-cell mass. This was associated with elevated serum insulin levels at GD9. ** p < 0.01, *** p < 0.001 *vs*. control (A and B), * p < 0.05, GD9 and GD18 *vs*. control (C and D). One-way ANOVA with Tukey’s post-hoc test, n = 4 per time point.

Maternal body weight increased steadily until GD18 confirming normal litter growth (Fig 1A). Beta-cell mass in the mother increased incrementally in response to the relative insulin resistance of pregnancy by approximately three-fold by GD18, and had rapidly regressed by 7 days post-partum (Fig 1B). IPGTT were performed at GD9 and 18 (Fig 1C). Compared to non-pregnant age-matched mice, the glucose excursions were significantly higher at GD9. To counter the emerging glucose intolerance serum insulin levels were significantly increased at GD9 compared to non-pregnant controls, but not at GD18 when the adaptive increase in ß-cell mass was maximal (Fig 1D). Thus, we validated the increasing maternal insulin resistance, compensatory increase in ß-cell mass and insulin release in the pregnant mouse.

### Proportion of beta-cell clusters increase in early pregnancy

**Fig 2:**
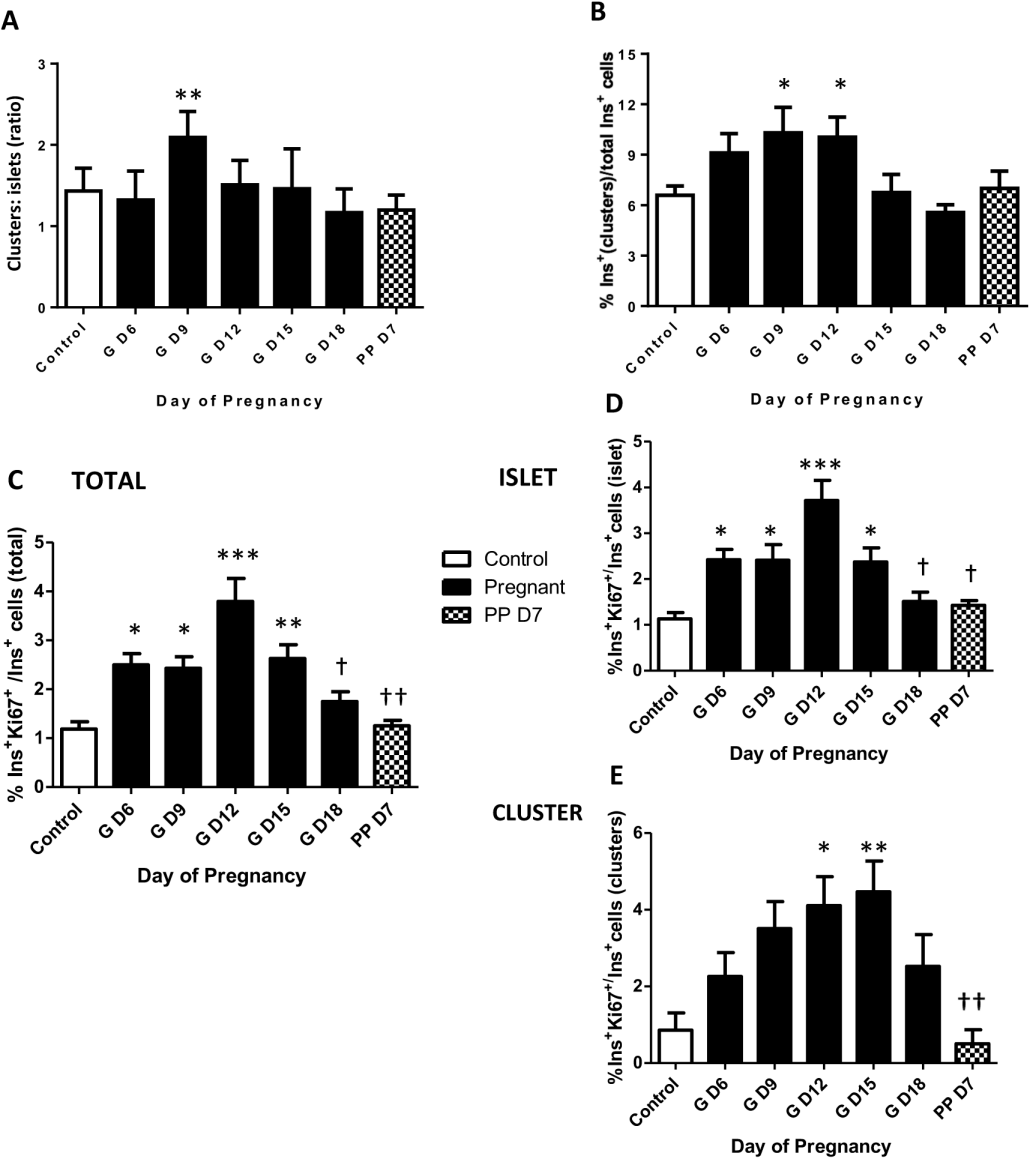
**Ratio of the number of β-cell clusters relative to islets (A), the proportion of β-cells present in clusters relative to the total population of β-cells (B), the percentage of total β-cells undergoing DNA synthesis (Ki67^+^) (C) and when separated into β-cells located in either islets (D) or clusters (E) in mouse pancreas between gestational days (GD) 6 and 18, and at postnatal day 7.** During pregnancy, the Ins^+^ cell population in β-cell clusters showed a greater proportional increase than in islets. The number of proliferating β-cells was greatest at GD12 and proliferating cells were located both in clusters and in islets. One-way ANOVA with Tukey’s post-hoc test, * p < 0.05, ** p < 0.01, ***p < 0.001 *vs*. control; † p<0.05, †† p<0.01 *vs*. GD 12 (n = 4 per time point).

**Fig 3:**
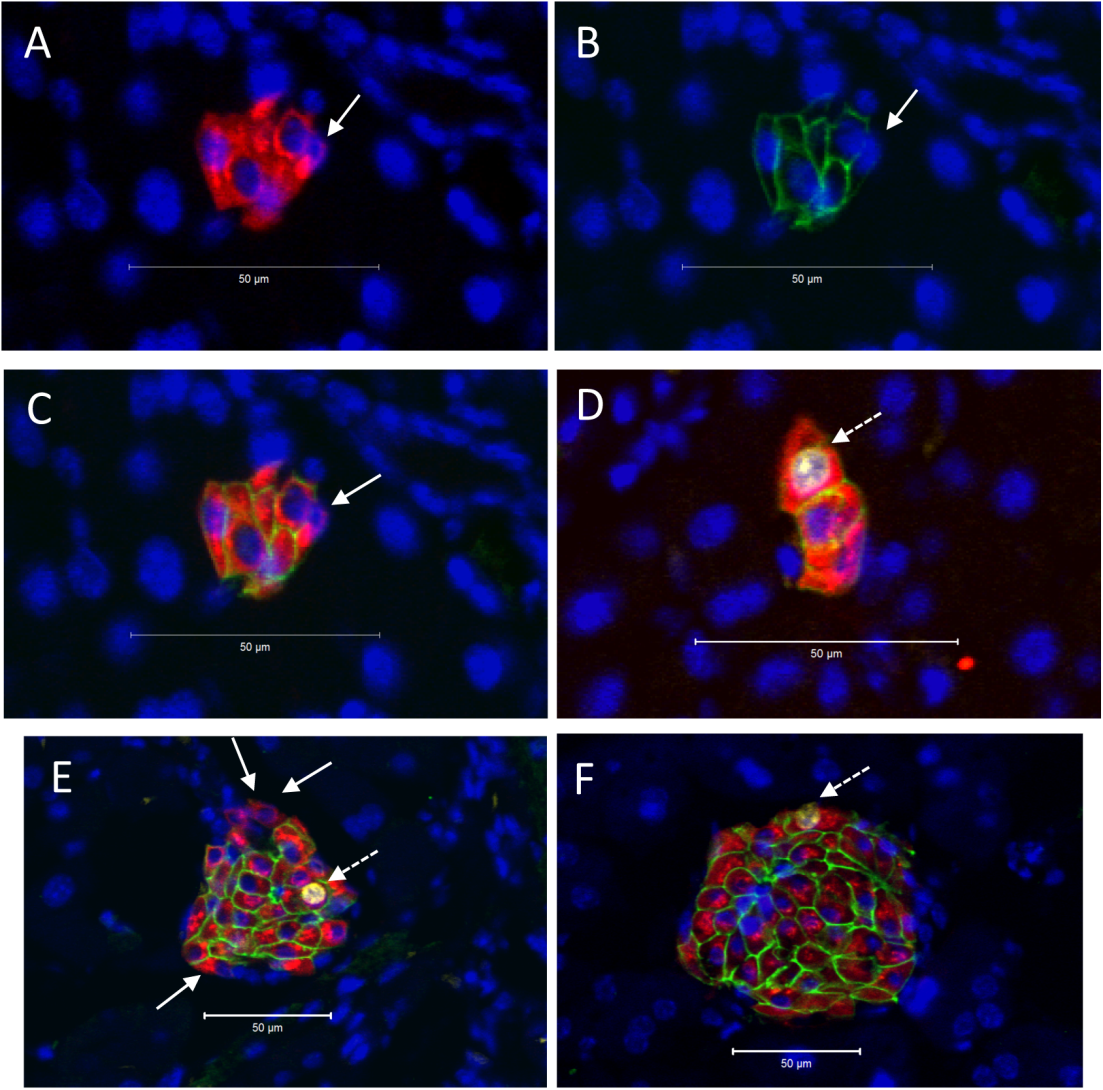
**Presence of β-cell clusters (A -D) and islets (E & F) in mouse pancreas at gestational day (GD) 9.** Immunohistochemistry was used to localize insulin (red), Glut2 (green), and Ki67 (white), whilst nuclei were counter-stained with DAPI (blue). Panels A-C show the same cluster to demonstrate insulin (A), Glut2 (B) and the merged image (C). Ins^+^Glut2^LO^ cells within clusters or islets without nuclear staining for Ki67 are indicated by the solid arrows in A-C and E. An Ins^+^Glut2^LO^ cell within a cluster showing nuclear staining for Ki67 is shown by the dashed arrow in D. Ins^+^Glut2^HI^ cells immunopositive for both Ki67 and Glut 2 can be seen in E and F (dashed arrows).

We previously reported that in young mice a population of multi-lineage potential cells existed that expressed low levels of insulin but lacked functional levels of Glut2, exhibited a higher rate of proliferation than mature β-cells, and were preferentially located in small, extra-islet endocrine cell clusters within the pancreas [22]. Therefore, we examined the relative abundance of insulin-expressing β-cell clusters throughout pregnancy A significant increase in the ratio of the number of clusters to islets was observed early in pregnancy at GD9, but not thereafter (Fig 2A). The percent of insulin-immunoreactive pancreatic cells associated with clusters compared to islets was also greater on GD12 (Fig 2B), and subsequently declined to values in non-pregnant mice. Examples of β-cell clusters are shown in Fig 3A–3D, containing both Ins^+^Glut2^HI^ cells and Ins^+^Glut2^LO^ cells (arrows). The Ins^+^Glut2^LO^ cells were also located within islets, but were less abundant than within clusters (Ins^+^Glut2^LO^ cells, islets vs clusters GD9, ** p < 0.01, t-test, not shown) (Fig 3E, arrows). These results suggest that a transient increase in the number of β-cell clusters occurs in early-mid-gestation with a consequent shift in the number of β-cells associated with clusters rather than islets. The transient nature of the increase in cluster number is likely to represent the consequent growth of these structures to become islets, which would therefore no longer fall within our definition of a cluster. Support for this possibility was sought by estimation of the proliferation rate of β-cells within clusters *vs*. islets.

### Beta-cell proliferation throughout pregnancy

We used immunohistochemical staining for Ki67 to identify insulin-containing cells that were undergoing DNA synthesis within whole pancreas, within islets or within β-cell clusters. Proliferating insulin-expressing cells expressing Ki67 were identified in both clusters and islets, and sub-populations of both Ins^+^Glut2^LO^ and Ins^+^Glut2^HI^ cells demonstrated nuclear staining for Ki67 (Fig 3). Within pancreas as a whole, the percent of β-cells undergoing DNA synthesis was approximately 1% in the non-pregnant state, but significantly increased as early as GD6 and was greatest on GD12 at around 4%, before a significant decline towards term (Fig 2C). Beta-cell proliferation had returned to non-pregnant values within a week of parturition. A similar profile was seen for β-cell proliferation within islets alone (Fig 2D). However, within clusters the percent β-cell proliferation remained elevated at GD15 (islet vs clusters, p < 0.05, t-test) and did not significantly decline until after parturition (Fig 2E). These data show that the expansion of β-cell mass through cell proliferation occurs from β-cells located within clusters as well as those within islets, and that the contribution from clusters is maintained as pregnancy progresses.

### Increase in Ins^+^Glut2^LO^ cell proportion precedes total β-cell expansion

**Fig 4:**
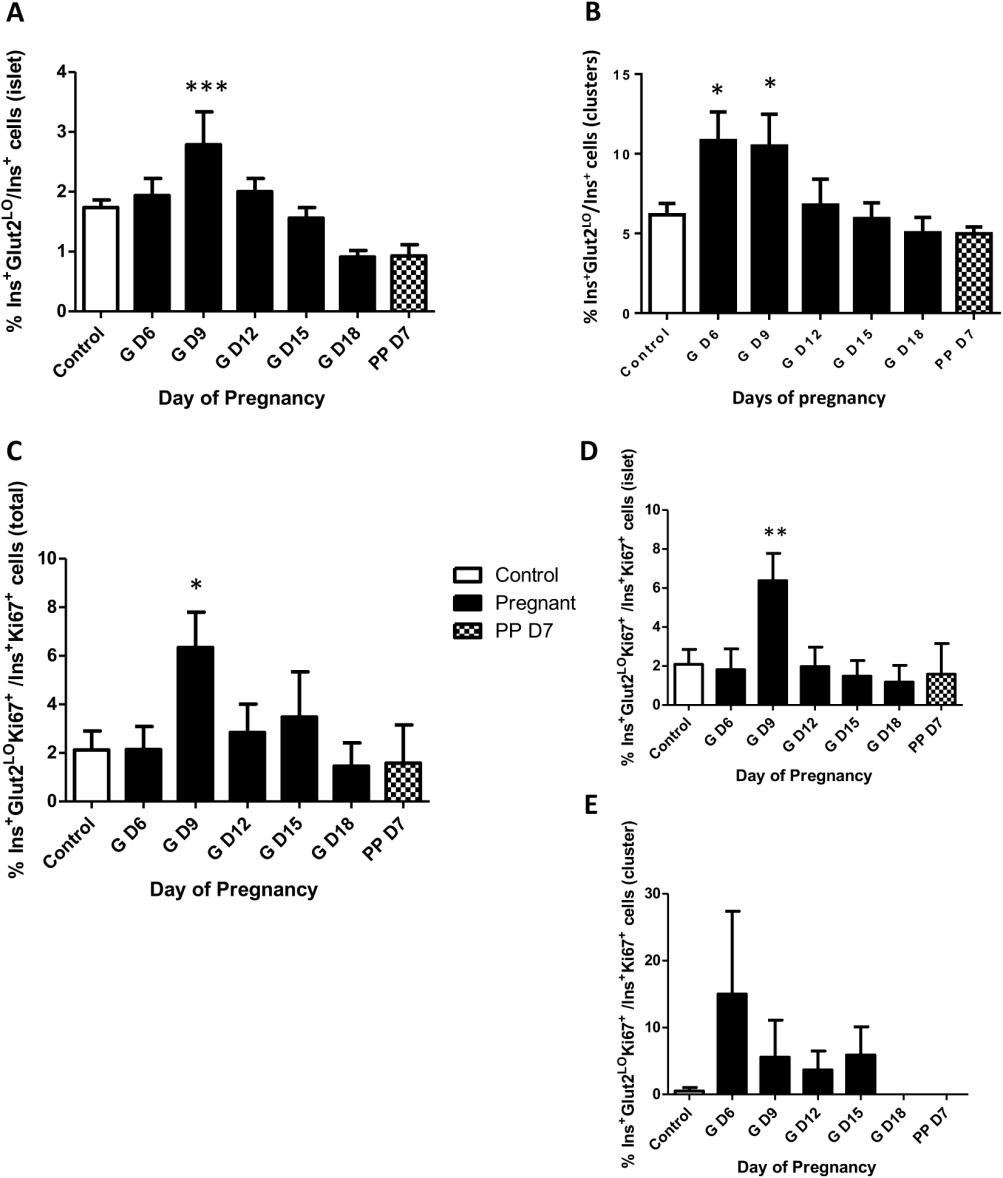
**Percent Ins^+^Glut2^LO^ cells within islets (A) or β-cell clusters (B) relative to total Ins^+^ cells within mouse pancreas between gestational days (GD) 6 and 18, and at postpartum (PP) day 7; and percent of proliferating (Ki67^+^) Ins^+^Glut2^LO^ cells within total pancreas (C), islets (D) or clusters (E) relative to all Ins^+^ Ki67^+^ cells.** The frequency of proliferation in Ins^+^Glut2^LO^ cells was increased 3-fold on GD9 within islets when compared to controls, subsequently declining in later gestation, * p < 0.05, ** p < 0.01, ***p< 0.001 *vs*. control (n = 4 per time point). One-way ANOVA with Tukey’s post-hoc test.

In order to directly address the contribution of Ins^+^Glut2^LO^ cells to β-cell expansion during pregnancy we calculated the percent Ins^+^Glut2^LO^ cells compared to all Ins^+^ cells within either islets or clusters. In both tissue compartments the percent contribution of Ins^+^Glut2^LO^ cells was significantly greater than in non-pregnant mice from GD6 in clusters and GD9 in islets (Fig 4A and 4B). This preceded the maximal increases in ß-cell mass expansion at GD18, and β-cell proliferation at GD12. We subsequently calculated the percent Ins^+^Glut2^LO^ undergoing DNA synthesis based on the presence of Ki67 in total endocrine pancreas, islets or clusters relative to all Ki67^+^Ins^+^ cells. A significant three-fold increase in Ins^+^Glut2^LO^ cell proliferation was seen at GD9 in pancreas as a whole and in islets, which subsequently declined with advancing gestation (Fig 4C and 4D). In clusters the percent proliferating Ins^+^Glut2^LO^ cells was highly variable between clusters (Fig 4E), peaking at GD6, before disappearing completely by late gestation and in the early post-partum time period. These findings suggest that on a cell-by-cell basis there is a greater contribution to β-cell proliferation from Ins^+^Glut2^LO^ cells than from mature β-cells, and that this commences early in gestation. Collectively, these data indicate that β-cell expansion during pregnancy occurs via multiple routes, including proliferation. Furthermore, the increase in β-cell mass can be attributed in part to an increase in Ins^+^Glut2^LO^ cell proportion, preceding the large increase in proliferation.

### Transcription factor expression levels within the pancreas during pregnancy reflect the generation of new β-cells, consistent with the increase in Ins^+^Glut2^LO^ cell proportion

We examined whether the expansion of maternal β-cell mass during pregnancy was correlated with changes in expression of transcription factors known to be associated with the lineage-commitment, proliferation, and functional maturation of β-cells during early life. Messenger RNAs were quantified using qPCR in whole pancreas from non-pregnant animals and at GD9 and 18. Whole pancreas was utilized so as to include the contribution from small β-cell clusters which would be otherwise excluded using standard islet isolation techniques. The expression of Pdx1 was significantly increased on GD9 relative to non-pregnant mice, but had returned to control levels by GD18 (Table 2). Levels of Nkx6.1 and MafB were significantly elevated at GD18.

**Table 2:** Fold changes in mRNA expression for transcription factors, at gestational days (GD) 9 and 18 relative to pancreas from non-pregnant female mice.

	GD9	GD18
Pdx1	2.14±0.99*	1.31±0.46
MafB	1.08±0.30	7.63±5.40*
Nkx6.1	3.51±0.71	2.32±0.62*

Values show mean±s.e.m. for 3–4 replicate animals. Mann-Whitney test

*p<0.05 *vs*. non-pregnant mouse

**Fig 5:**
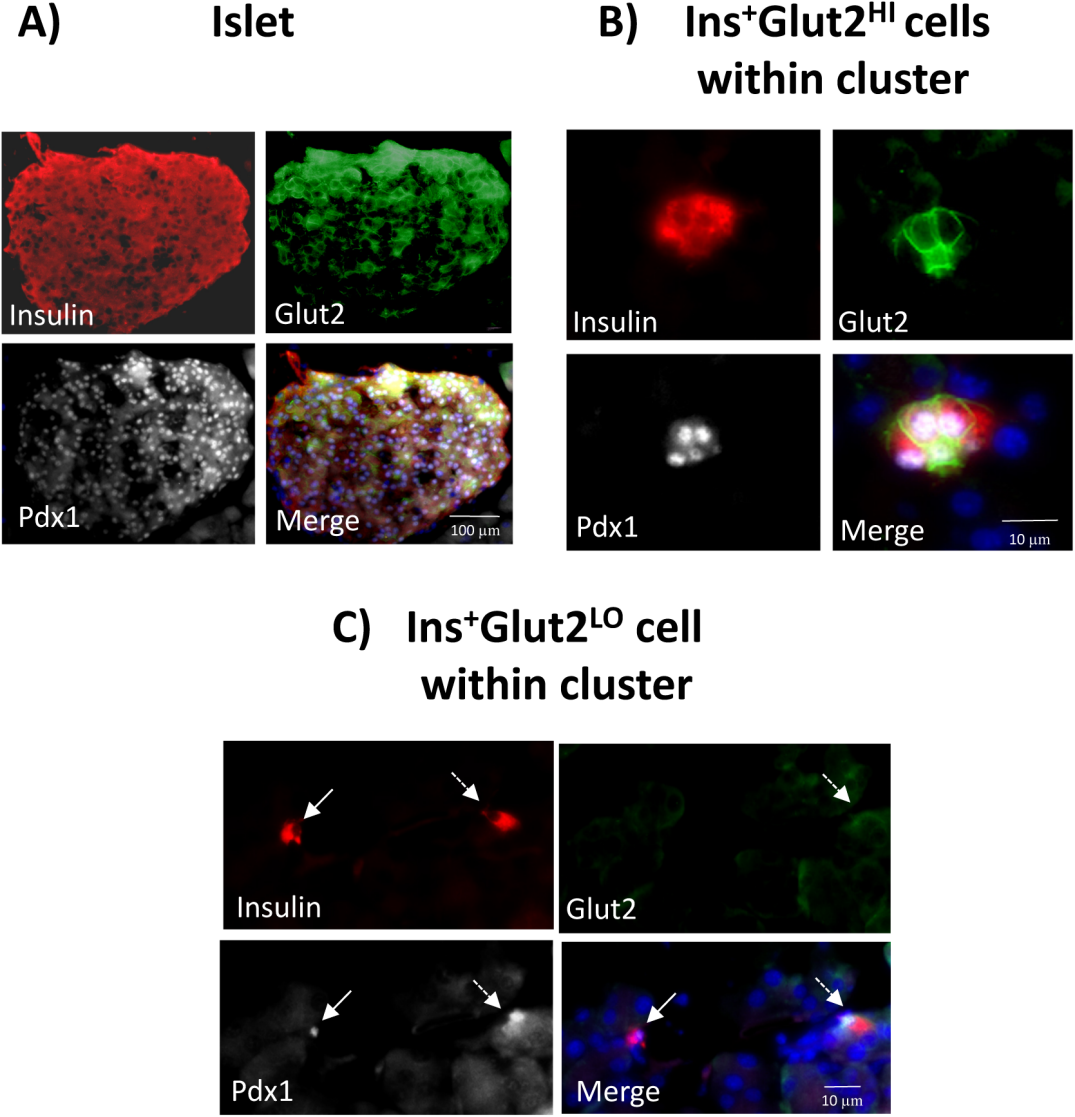
Co-localization of Pdx1 (white) with insulin (red) or Glut2 (green) in mouse pancreas at gestational day (GD) 9. Nuclei were counter-stained with DAPI (blue). An islet of Langerhans is shown in A, and small β-cell clusters in B and C. Panel B shows a cluster containing Ins^+^Glut2^HI^ cells demonstrating the presence of nuclear Pdx1together with cytoplasmic insulin and Glut2 associated with the plasma membranes. Panel C shows an Ins^+^Glut2^LO^ cell (solid arrow) within a small cluster containing nuclear Pdx1as well as cytoplasmic insulin, but lacking detectable Glut2. To the right is shown an Ins^+^ cell with detectable Glut2 and nuclear-localized Pdx1(dashed arrow).

We further determined if Pdx1 protein was present within the nuclei of Ins^+^Glut2^LO^ cells, especially within small β-cell clusters, at the time of β-cell expansion. Immunohistochemistry showed that the majority of Ins^+^Glut2^HI^ cells within the islets at GD9 contained Pdx1, as would be expected for functional β-cells also containing insulin and Glut2 (Fig 5A). Similarly, Ins^+^Glut2^HI^ cells within small β-cell clusters demonstrated nuclear Pdx1 localization (Fig 5B), as did a sub-population of Ins^+^Glut2^LO^ cells that contained insulin, but not Glut2 (Fig 5C). We attempted to quantify changes in Pdx1 protein in pancreas during pregnancy by measuring the Pdx1 content in pancreata from non-pregnant mice, at GD9 and GD18 using an ELISA. The Pdx1 presence in pancreas from age-matched non-pregnant mice was 0.24±0.02 ng/μg protein, compared to 0.30±0.06 ng/μg at GD9, and 0.46±0.01 ng/μg at GD18 (n = 3–4), but these values did not differ significantly.

## Discussion

As has been reported previously [26], β-cell mass steadily increases in the pregnant rodent and was three-fold higher on GD18 compared to age-matched, non-pregnant animals, before regressing within seven days post-partum. A significant glucose intolerance compared to non-pregnant mice was observed at GD9, but not so at GD18 when the adaptation of β-cell mass was complete. Also, in confirmation of previous findings [2], the increase in β-cell mass was accompanied by greater than a doubling in proliferation rate of β-cells within the islets which was maximal on GD12, in advance of the maximal increase in β-cell mass.

Our results show that the expansion of β-cell mass during pregnancy arises not only from the renewed proliferation of β-cells within the anatomical islets, but also from those within the abundant extra-islet endocrine clusters. The relative number of such clusters relative to islets was increased in the first half of gestation, as was the percentage of β-cells within clusters and the sub-population of these undergoing proliferation. This suggests that new clusters are being formed during pregnancy, either by pancreatic neogenesis or by the proliferation of isolated, resident endocrine progenitor cells within the pancreas. The subsequent decline in the number of clusters, despite a continuing proliferation of ß-cells most likely indicates their continued volume expansion such that once they become small islets (>5 β-cells) they were no longer recorded by us as clusters. This is in agreement with the findings that the number of islets per unit area of pancreas also increases in the second half of mouse pregnancy [10]. Our results would suggest that the formation of new clusters represents a developmental wave of neogenesis that does not continue into later pregnancy, presumably since an adequate homeostatic pathway leading to functional β-cells has been established. This is further suggested by the loss of proliferating Ins^+^Glut2^LO^ β-cells at the end of pregnancy and in the early postpartum time period, specifically within clusters.

An Ins^+^Glut2^LO^ population of β-cells has been identified as putative, resident progenitors of mature functional β-cells [19, 20]. Such cells have pancreatic endocrine multi-potentiality and, unlike most mature β-cells, are proliferative. They are retained in small numbers in adult life and have been implicated in the regeneration of β-cells in mice following the experimental reduction of β-cell mass [27]. As reported by us previously [22], the Ins^+^Glut2^LO^ cells were most abundant in extra-islet β-cell clusters. The percentage of Ins^+^Glut2^LO^ cells relative to total β-cells was elevated within clusters as early in pregnancy as was examined, at GD6, but declined to values found in non-pregnant animals by GD12. As mentioned above, this decline is also likely to be artefactual as clusters expanded to become small islets and were no longer identified as clusters. Within clusters, the percent of Ins^+^Glut2^LO^ cells undergoing DNA synthesis was increased but highly variable between clusters early in pregnancy when compared to the non-pregnant state. Recent studies by Bader et al. [28] used the genetic marker of terminal differentiation and polarization of β-cells, *Fltp*, to distinguish largely non-proliferative, mature β-cells in adult mouse pancreas from a minor population of immature but proliferative β-cells within islets. The *Fltp*-negative β-cells were shown to have a substantially differing gene expression profile, including a relative lack of *Slc2a2* (Glut2). Of relevance to the present study, the abundance of *Fltp*-negative β-cells and the percentage of these that were proliferating was greater in pancreas from pregnant mice at GD15.5 than from non-pregnant animals, and could be demonstrated to mature into *Fltp*-positive β-cells that subsequently expressed other markers of maturation such as the transcription factor *Nkx6*.*1*. A recent paper further confirmed the presence of Ins^+^Glut2^LO^ cells in the non-pregnant mouse and human pancreas, particularly at the periphery of the islet structures [29]. It was concluded that many of these cells were immature β-cell progenitors that had derived from the de-differention of alpha cells, and that these cells contributed to metabolic adaptations of β-cell mass.

We observed that in whole pancreas the levels of mRNA for the transcription factor Pdx1 were significantly increased during pregnancy at GD9, but had returned to non-pregnant values by GD18. Since *Pdx1* is expressed during the differentiation of β-cells from progenitors during early life [30] this would be consistent with a wave of neogenesis or progenitor maturation early in gestation giving rise to an increased abundance of extra-islet endocrine cell clusters. We confirmed that at GD9 a sub-population of Ins^+^Glut2^LO^ cells within clusters contained nuclear-associated Pdx1 using immunohistochemistry, and as would be expected for functional β-cells, so did Ins^+^Glut2^HI^ cells. The *Ngn3*-expressing cells were found mainly in the acinar tissue during pregnancy rather than ducts or islets, supporting the concept of a maturation of resident β-cell progenitors predominantly outside of the islets during gestation. We also observed an increase in the expression of *Nkx6*.*1* at GD18, consistent with an expansion of the immature β-cell population and their subsequent maturation. *MafB* expression was also relatively increased at GD18. The expression of *MafB* is associated with β-cell immaturity in the developing pancreas but is largely restricted to pancreatic alpha-cells in adult mice [31]. However, during pregnancy, a sub-population of *MafB*-expressing β-cells appeared within islets although these were not typically shown to be proliferative [32]. On the other hand, deletion of the *MafB* gene from β-cells, or deletion of the prolactin receptor which was shown to transcriptionally control *MafB*, resulted in a failure of adaptive expansion of β-cell mass during pregnancy, and the development of gestational diabetes [33]. This strongly suggests that the expression of *MafB* is a feature of a resident progenitor β-cell population that also expresses *Glut2* poorly, is proliferative during early pregnancy, and can contribute to an expanding mass of β-cells. Since the expression of *MafB* was significantly higher in late gestation pancreas than in non-pregnant mice it is possible that the newly-generated β-cells are still functionally immature. It is challenging to select an appropriate housekeeping gene with which to calibrate changes in transcription factor expression by qPCR within pancreas during pregnancy, as the tissue must be processed rapidly to avoid protein degradation and the endocrine tissue remodeling is substantial. We used both GAPDH and cyclophilin and employed a parallel amplification method to show appropriate amplification between the housekeeping gene and the gene of interest. Also, animals were killed at the same time of day during the inactive daylight period for mice when substantial excursions in blood glucose would not be expected.

Our findings provide evidence for the contribution of resident Ins^+^Glut2^LO^ cells to β -cell expansion as a physiological response to pregnancy, and suggest that the extra-islet β-cell clusters are an important source of new β-cell formation and maturation. However, the absence at present of an Ins^+^Glut2^LO^ cell-specific positive gene expression marker precludes the specific lineage tagging of these cells to confirm their eventual phenotypic status within the β-cell population of pregnant mouse pancreas. Also, the fate of these cells following parturition is unknown, and whether the β-cells derived from Ins^+^Glut2^LO^ progenitors are preferentially lost or retained as β-cell mass is reduced through apoptosis remains elusive. Finally, the contribution of an altered number or proliferation of Ins^+^Glut2^LO^ cells to the inadequate β-cell mass associated with gestational diabetes requires further study.
